# Supplementary material for: Phenotypic characterization of primary cardiac fibroblasts from patients with HFpEF
Source: PLoS One. 2022 Jan 11;17(1):e0262479. doi: 10.1371/journal.pone.0262479 (PMC8752005; doi:10.1371/journal.pone.0262479)
Supplement: S1 Fig — Values were normalized to levels of tubulin. A trend toward greater levels of collagen I in cell layers from HTN(+)HF fibroblasts was observed. Fibroblasts from P2 or P3 were used for analysis. Referent control (n = 4), HTN (-) HFpEF (n = 4), HTN(+) HFpEF (n = 6). (PPTX) [file pone.0262479.s001.pptx]

## Slide 1
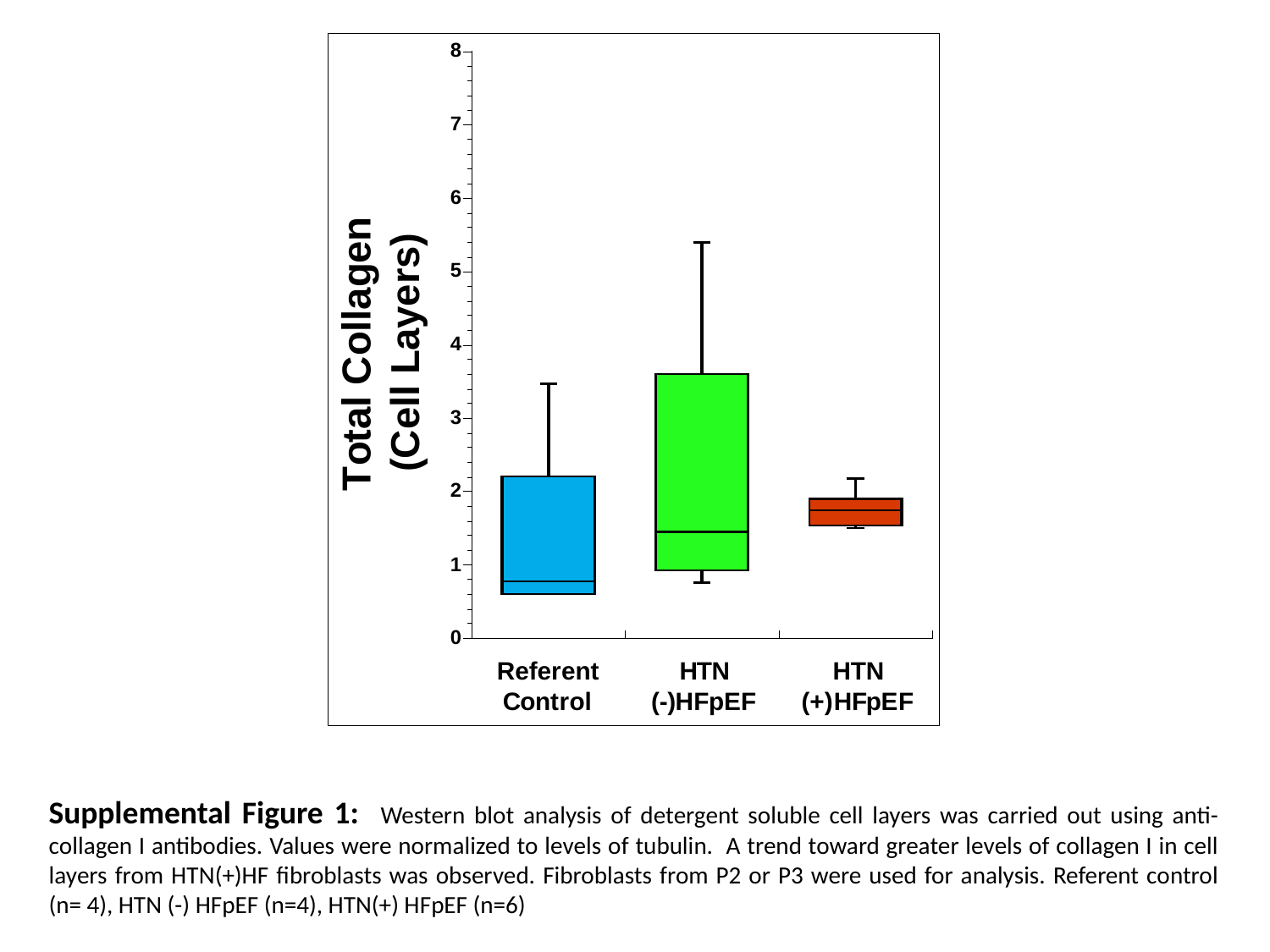

Supplemental Figure 1: Western blot analysis of detergent soluble cell layers was carried out using anti-collagen I antibodies. Values were normalized to levels of tubulin. A trend toward greater levels of collagen I in cell layers from HTN(+)HF fibroblasts was observed. Fibroblasts from P2 or P3 were used for analysis. Referent control (n= 4), HTN (-) HFpEF (n=4), HTN(+) HFpEF (n=6)
