# Supplementary material for: Phenotypic characterization of primary cardiac fibroblasts from patients with HFpEF
Source: PLoS One. 2022 Jan 11;17(1):e0262479. doi: 10.1371/journal.pone.0262479 (PMC8752005; doi:10.1371/journal.pone.0262479)
Supplement: Attachment — Submitted filename: Response to Reviewers.docx [file pone.0262479.s002.docx]

We thank the editors and the reviewers for the helpful comments that improved our manuscript. We have addressed each reviewer concern individually below. As per journal instructions, we have 1) addressed PLOSOne style requirements, 2) clarified in our Methods that *written consent* was obtained from study participants, 3) included in the cover letter a repository URL containing blot images as raw data images, 4) included a full ethics statement in the ‘Methods’ section as requested, and 5) Fig. 3 is now cited in the text.

***Author’s Responses to Questions:***

Reviewer #1:

1- As the authors describes, heart failure with a preserved ejection fraction (HFpEF) is characterized with an important increase in myocardial fibrosis. In the Figure 1, the authors show Picro Sirius representative images, the reference control and HTN (-) HFpEF evidence more interstitial fibrosis, in contrast the HTN (+) HFpEF image seems to present interstitial and perivascular fibrosis. The authors should specify whether there are differences in interstitial and perivascular fibrosis for the three groups showing images of interstitial/perivascular fibrosis. In addition, if there are differences or not you should discuss about their pathological meaning.

*The reviewers make a valid point concerning vascular versus perivascular fibrosis. However, we are, unfortunately, somewhat limited by the size of the epicardial biopsies. After the biopsies are divided for immunohistochemistry and for fibroblast preparations, the size of the resulting tissue sections used to evaluate IHC are small. In some cases, large or medium vessels are present in the biopsies but not for each. Thus, we did not include perivascular versus interstitial fibrosis as a parameter as we are not able to make this determination equally for each biopsy recovered. We have added this point to the Discussion.*

To this point, determinations of perivascular versus interstitial fibrotic deposition was not performed as sections from each biopsy did not necessarily contain equal vascularity in terms of blood vessel size and number.

2- The authors calculate the collagen volume fraction according to Bradshaw, et al., 2009. As I have already commented above, due to the importance of the fibrotic process in HFpEF pathology, you should explain the collagen measurements in more detail (Figure 2). How many images you quantify for each group? Do you quantify different sections for each patient?

*We have now included in the Methods section more details of this quantification as requested, only one biopsy was collected for each patient. Five regions of interest in three separate sections were evaluated per patient.*

One anterior free wall epicardial - midwall biopsy ( ~20-100) mgs was collected from each patient during intraoperative procedures after the patient was placed on cardiopulmonary bypass.

Each biopsy was viewed under polarized light to capture the birefringence generated by the fibrillar collagen present in the section. Collagen volume fraction (CVF) was then calculated from five separate regions of interest (RIO) from three separate sections taken from each biopsy using SigmaScan Pro and presented as percent collagen content of the total area in the ROI [8].

3- Please, explain in more detail the quantification of myocyte cross sectional area (how many fields of each sample).

*We have added in the Methods a more detailed explanation of the quantification including the number of myocytes assessed in the biopsies from the different groups to reflect the robustness of the count.*

Wheat germ agglutinin conjugated to peroxidase (Vector Labs) was incubated on myocardial sections and detected using tyramide amplification (Biotium). Stained sections were visualized on a Keyence BZ-X800 fluorescent microscope which allows for reconstruction of stitched images such that the entire region of the tissue section can be viewed in one image. Three fields per biopsy were evaluated that contained myocytes with optimal cross-sectional orientation, approximately 500 cells per field were quantified (~1500 cells per biopsy). One section per biopsy was used to evaluate CSA. Myocyte cross sectional area (CSA) was calculated using the Hybrid Cell Count function (Keyence BZ-X800 Analyzer).

4- The authors mention that they have observed no significant changes in cardiac fibroblast proliferation, but this may be can due to the low concentration of FCS (2%) used to stimulate proliferation. It is commonly used between 5-10% FCS to stimulate fibroblast proliferation (Lopez-Martinez, et al., 2019; Cañes L, et al., 2020). Moreover, the reference that the authors mention uses 10% FCS to stimulate fibroblast (Figure 4 of Bradshaw, et al., 1999). Do the authors analyze the impact of high FCS concentrations on proliferation assays?

*We thank the reviewers for their expertise and insight. At the time these studies were initiated, we established that 2% and 5% FCS evoked similar increases in proliferation as measured by ^3^H thymidine incorporation and hence we adopted 2% FCS as our standard concentration to use across preparations to induce proliferation from baseline levels (BSA only). In addition, our standard growth media for propagating the primary human fibroblast cultures contains 10% FCS. Notably, we have not observed significant differences in proliferative capacity associated with our designated groupings in standard culture with 10% FCS. Hence, we have concluded that neither 5 or 10 % FCS concentrations would likely yield significant differences in proliferation across the three groups versus that observed with 2% FCS. However, we cannot rule out the possibility that 10% FCS might induce greater changes in proliferation from baseline in the ^3^H Thymidine assay, as we did not test this parameter directly. At this point, we are not able to retroactively perform the experiments with 10% FCS.*

5- Due to the partly insoluble character of collagen, western blotting analyses are not a reliable tool to determine the collagen production. The authors must confirm their western blotting results with other technics.

*We thank the reviewers for this comment as we also appreciate the importance of assessing separate cell compartments for a complete analysis of collagen production and deposition. To this point, we evaluated levels of procollagens and collagen in both conditioned media and in deoxycholate detergent-soluble cell layers. We now note in the text that similar differences in levels of collagen were also observed in blots performed with cell layers and show the graph as a newly added Supplemental Fig. 1.*

*As to the reviewer’s point with respect to alternate methods for collagen detection, in our experience the amount of detergent (deoxycholate)-insoluble collagen deposition in the relatively short time frame (2-4 days) is not measurable by hydroxyproline analysis or radio-labeled collagenase assays. In fact, we did not detect collagen bands in deoxycholate insoluble pellets boiled in SDS-Laemmli buffer. We have found the Sircol assay unreliable for collagen detection in vitro. In our experience, western blot detection of collagen in detergent soluble layers and conditioned media for fibroblast cultures of this length recover the majority of collagen produced by fibroblasts in these conditions.*

6- Please, improve the tubulin western blotting in figure 4 and 5.

*We appreciate the reviewer’s comment and agree that the quality of the tubulin western blot in Fig. 5 was substandard. We have now provided a new Fig. 5 containing an improved tubulin western blot for these samples. Unfortunately, sufficient amounts of sample were not available to repeat the tubulin blots from Fig. 4, however the quality of this blot is better than that of the original in Fig. 5.*

Reviewer #2: 
Major concerns:
- My main concern is regarding the data obtained in the primary cell culture experiments. It has been shown (Cardiovasc Res. 2007 Aug 1;75(3):519-29; J Pathol. 2019 May;248(1):30-40.) that in primary fibroblast cell culture cells at passage 1 are regular fibroblast whereas cells at passage 3 are considered activated fibroblasts. Since here, some experiments were performed at passage 1 and others at passage 2 or 3, the results and experiments should be revised.

*The reviewer raises an important point which we would like to address as we have considered this issue ourselves. First, we find that the number of fibroblasts generated from the relatively small biopsies at P0 are not sufficient to yield enough cells to perform assays for proliferation and protein production. Hence, by necessity, cells must be passaged to yield numbers required to perform these assays. Cells were used at P1 - P3 to generate consistent reproducible data: proliferation assays were carried out at P1 or P2, and protein production at P2 or P3. The reviewer provided two references in which neonatal rat cardiac fibroblasts are shown to progress to a myofibroblastic phenotype from P1 to P3. We have not found a similar αSMA increase in our adult human cardiac fibroblast preparations. Although we have not followed this parameter for each isolation, our initial studies did not find substantial increases in αSMA between passages 1 and 3. Our results are supported by those of Flack et al. using a large animal porcine model of left ventricular failure in which fibroblasts isolated from normal and failure pigs demonstrated distinct, consistent differences from P1 through P4 that did not progress into a myofibroblastic phenotype with passage (JMCC, 2006, 40: 474-483). We would also point out the report from Jonsson et al. in which fetal and adult human cardiac fibroblasts were compared by transcriptional analysis. Adult human cardiac fibroblasts were used in passage 2 and 3 and were found to have minimal αSMA production with 98% being characterized as αSMA negative (JACC Basic to Translational, 2016, Vol. 1, 7). As shown in Fig. 6, we monitored aSMA production in cells from each group over time at P3 and did not find consistent differences or increases in aSMA production over days in culture. Admittedly, we cannot rule out that culturing fibroblasts on tissue culture plastic introduces a non-physiological tensile strength that masks certain phenotypic differences between the different groups. For this reason, we included the culture conditions as a limitation in the Discussion.*

- Related to this, the passage of the cells should be clearly stated in figure legends and in the text.

*Thank you for this recommendation, we now clearly state the passages in the Figure Legend and text (methods).*
